# The relative importance and stability of disease burden causes over time: summarizing regional trends on disease burden for 290 causes over 28 years

**DOI:** 10.1186/s12963-021-00257-0

**Published:** 2021-06-10

**Authors:** Henry Dyson, Raf Van Gestel, Eddy van Doorslaer

**Affiliations:** 1 Erasmus University Rotterdam, Rotterdam, The Netherlands; 2 Erasmus School of Health Policy and Management & Erasmus School of Economics, Erasmus University Rotterdam, Rotterdam, The Netherlands

**Keywords:** Burden of disease, Trends, Summary statistic, Gini

## Abstract

**Background:**

Since the Global Burden of Disease study (GBD) has become more comprehensive, data for hundreds of causes of disease burden, measured using Disability Adjusted Life Years (DALYs), have become increasingly available for almost every part of the world. However, undergoing any systematic comparative analysis of the trends can be challenging given the quantity of data that must be presented.

**Methods:**

We use the GBD data to describe trends in cause-specific DALY rates for eight regions. We quantify the extent to which the importance of ‘major’ DALY causes changes relative to ‘minor’ DALY causes over time by decomposing changes in the Gini coefficient into ‘proportionality’ and ‘reranking’ indices.

**Results:**

The fall in regional DALY rates since 1990 has been accompanied by generally positive proportionality indices and reranking indices of negligible magnitude. However, the rate at which DALY rates have been falling has slowed and, at the same time, proportionality indices have tended towards zero. These findings are clearest where the focus is exclusively upon non-communicable diseases. Notably, large and positive proportionality indices are recorded for sub-Saharan Africa over the last decade.

**Conclusion:**

The positive proportionality indices show that disease burden has become less concentrated around the leading causes over time, and this trend has become less prominent as the DALY rate decline has slowed. The recent decline in disease burden in sub-Saharan Africa is disproportionally driven by improvements in DALY rates for HIV/AIDS, as well as for malaria, diarrheal diseases, and lower respiratory infections.

## Introduction

**Fig. 1:**
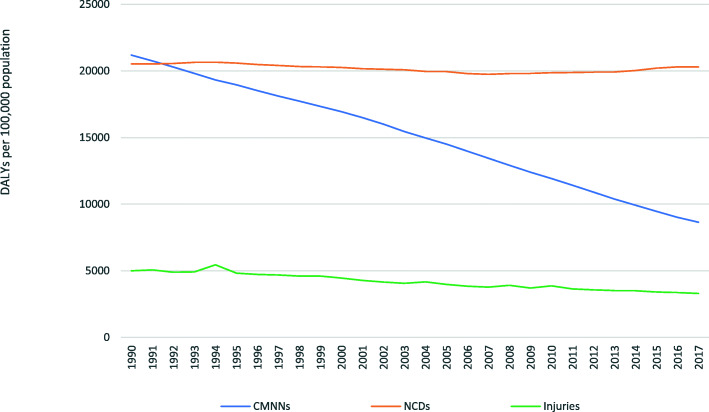
Global DALY rates per 100,000 by broad CoD (causes of disease) burden, 1990–2017

By grouping causes of death as ‘communicable, maternal, neo-natal and nutritional diseases (CMNN)’, ‘non-communicable diseases (NCDs)’, and ‘injuries’, Murray and Lopez [19] summarized findings on causes of death for eight regions of the world using data from the 1990 wave of the GBD study. Although NCDs were generally found to be the leading causes of death worldwide, five of the top ten leading causes of death were the result of CMNN diseases. Both the probability of dying from CMNN diseases and from NCDs was significantly higher in developing regions such as sub-Saharan Africa than in developed regions. Over two decades after this initial study, two NCDs, ischemic heart disease (IHD) and stroke, remain responsible for by far the largest number of global deaths [9]. CMNN diseases, especially pneumonia, neo-natal conditions, and diarrheal diseases, are still important causes of death, particularly in developing regions. However, these broad similarities mask a more complex picture of the varying relative importance of death causes. The importance of some global causes of ‘disease burden’, measured in the GBD using Disability Adjusted Life Years (DALYs)^1^, has changed substantially. An example which clearly demonstrates this variation is the increase from 18.6 to 29.8% of total DALYs attributable to NCDs in Sub-Saharan Africa between 1990 and 2017 [13]. The declining importance of CMNNs is also visible at the global level (see Fig. 1).

Health trends are continuously monitored and presented by international collaborators (among others, [9–11, 14, 28]), academics and governments (among others, [4, 7, 8, 20, 21, 24, 29, 32]).

However, deriving clear trend data from the GBD study is a challenging task because it requires summarizing data on a particular health metric across 28 possible years, 290 potential classifications of DALY causes for 195 countries and territories, i.e. for over a million data points. Recent publications (e.g. [9–11, 18, 28]) have addressed this problem in one of two ways: (i) by presenting data for all classifications but for only one or two selected years and/or locations, or (ii) by presenting trends over many years but only for selected causes, or very broad definitions of causes (e.g. CMNN diseases, NCDs, injuries). Due to the large number of classifications, the detailed appendices attached to the over 50-page GBD summary papers comprise close to 10,000 pages (e.g. [9]: Supplementary annex 2), and yet are still selective in the presentation of metrics and years.

We use two quantitative measures that summarize (1) whether, over time, the growing or declining overall DALY rates are disproportionally attributable to ‘major’ (e.g. Ischemic heart disease, stroke) or ‘minor’ (e.g. Ebola, osteoarthritis) DALY causes, and (2) whether there are substantial changes in the ranking of diseases in terms of severity. These two measures derive from a decomposition of the Gini coefficient. The Gini was originally developed to measure changes in income inequality and mobility. In this context, the Gini captures the degree to which the disease burden is more or less concentrated among disease causes.

For policymakers, the two measures provide a helpful extension to complement existing trend data on cause-specific DALYs by summarizing a large amount of data that may otherwise be hard to interpret. The first measure broadly informs on the relative importance of disease causes. This analysis over time could therefore form an instrumental part of the process of deciding whether resources should be reallocated in response to the changing relative importance of major or minor causes. Additionally, it is widely accepted that increasing uncertainty should lead to the diversification of risks. Hence, with rising uncertainty on the importance of DALY causes — the recent COVID-19 epidemic is a clear illustration of that — as reflected in the variability of the measures over time, it is wise to spread the allocation of resources across a variety of diseases (through, e.g. R&D expenditures). The summary measures also provide more food for thought on how to reallocate resources strategically (and by how much). For example, the stability in the absolute ranking of diseases may provide suggestive evidence that the prioritization of resources between different disease causes should also remain stable. Discussions on reallocation of attention and resources could be initiated by the WHO and the World Bank, as well as by national governments.

The paper proceeds as follows: the Methods and data section explains the foundations of the Gini coefficient and its decomposition. It also describes the data and outlines how the data analysis is presented. The Results section presents the results of the data analysis. Finally, the Discussion and limitations section addresses the limitations of this study and the Conclusion section concludes.

## Methods and data

### Gini coefficients

Measures of concentration such as the Gini coefficient have most frequently been used as tools to evaluate the degree of relative income or wealth inequality (e.g. [5, 17, 30]). However, Gini-like measures have also been applied in many other areas, including in health economics (e.g. [6, 25, 27]. In a recent article, Barrenho et al. [2] used data from the GBD to rank causes of DALYs by their respective contributions to the total number of global DALYs. They showed that Gini-like indices (i.e. the concentration index) can be used to estimate whether or not innovation is disproportionately concentrated in more highly ranked causes.

**Fig. 2:**
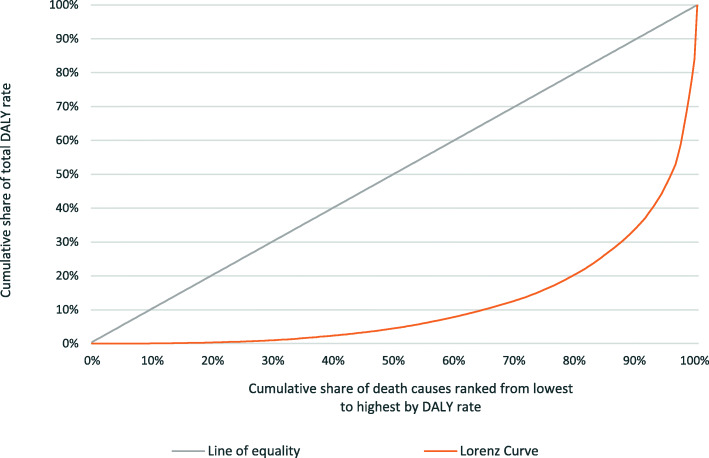
2017 Lorenz curve for 290 CoD burden ranked from lowest to highest by contribution to the global DALY rate

In a similar way, we make use of the rankings of the causes of DALYs, but the aim here is to instead understand to what extent DALY *rates* are disproportionately concentrated in high- versus low-ranked causes. To illustrate how this can be done, Fig. 2 displays a Lorenz curve which makes use of the global DALY rates for 290 causes of disease (CoD) burden in 2017. The causes are ranked from lowest to highest according to contributions towards the total DALY rate. The horizontal axis in Fig. 1 represents the cumulative share of the total number of disease burden causes, with the lowest ranked cause representing the first point on this axis and each point along the axis representing a more highly ranked cause.

The vertical axis shows the cumulative share of the total disease burden resulting from each cause. If all disease causes had equal shares of DALY rates, then the cumulative distribution would simply be a diagonal line, indicating perfect equality. In reality, Fig. 2 shows that in 2017, the 10% lowest ranked (29 out of 290) disease causes account for less than 1% of the total global DALY rate. By contrast, the 10% highest ranked disease burden causes were responsible for over 65% of the total global DALY rate in that year. That, as might be expected, signals a very unequal distribution of the disease burden.

The degree of inequality can be measured by a Gini coefficient defined as twice the area between the equality line and the Lorenz curve. The Gini is bounded between 0 and 1. A value that is close to 1 (0) indicates that the disease burden is more (less) concentrated in the major causes (see Appendix 1 for a mathematical expression of the Gini).

### A decomposition of the Gini coefficient

The Gini coefficient provides a fairly simple way to express the extent to which DALY rates are more or less concentrated in certain causes. It can also measure changes over time as a difference in Ginis (∆*G*) but the most interesting information can be obtained from decomposing this change into two parts. Jenkins and van Kerm [15] proposed to decompose the change in a Gini coefficient into a ‘Reranking’ and a ‘Proportionality’ component. Letting the subscripts 0 and 1 denote an earlier and later point in time, respectively, the decomposition of the change in the Gini can be shown to equal:

1 $$ \Delta  G\equiv {G}_1-{G}_0\equiv R-P, $$

where,

2 $$ R={G}_1-{G}_1^{(0)} $$

3 $$ P={G}_0-{G}_1^{(0)}. $$

*G*_0_ and *G*_1_ are the Gini coefficients in year 0 and year 1, respectively, and ${G}_1^{(0)}$ is the coefficient for year 1 DALY rates calculated according to year 0 ranks (this is then a concentration rather than a Gini index because the ranking variable is different from the quantity of interest). *R* is the change in the Gini coefficient that can be attributed to ‘reranking’ and *P* is the change in the Gini coefficient that can be attributed to ‘proportionality’.^2^ The proportionality index, *P*, can be defined as the change in the Gini coefficient that would have occurred if rankings had been held constant at their pre-distribution position.^3^

**Fig. 3:**
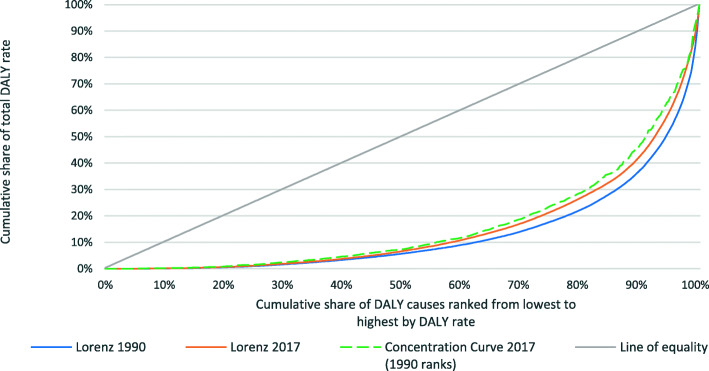
1990 and 2017 Lorenz curve for 290 DALY causes ranked from lowest to highest by contribution to global DALY rate in 1990 and 2017, respectively; 2017 concentration curve for 290 DALY causes ranked from lowest to highest by contribution to global DALY rate in 1990

Figure 3 illustrates this result graphically using the example of 1990 and 2017 global DALY rates. The inward shift of the Lorenz curve over the period shows that global DALY rates have become less concentrated in the leading causes over the period. This can especially be seen at the lower end of the distribution where a higher percentage of DALYs is accounted for by the minor causes. Twice the area between the Lorenz curves for 1990 and 2017 is the change in the Gini coefficient, ∆*G*. This change can be broken down into two parts. The first is the difference between the Lorenz curve for 1990 DALY rates and the concentration curve for 2017 DALY rates constructed using 1990 DALY rate ranks. This summarizes the ‘proportionality’ of the DALY rate reductions: −*P* is twice the area between these two curves. One way to interpret this value is that it is the *change in Gini coefficient that would have occurred had there been no change in the ranking*. The second component is the difference between this concentration curve and the Lorenz curve for 2017, which summarizes the extent of reranking. *R* is twice the area between these two curves. This value can be interpreted as the *change in the Gini coefficient in the most recent period that would occur if the ranking of diseases would have remained the same as the ranking in the earlier period.* The figure illustrates that the Gini has fallen in value over the period because *P* > *R*.

**Fig. 4:**
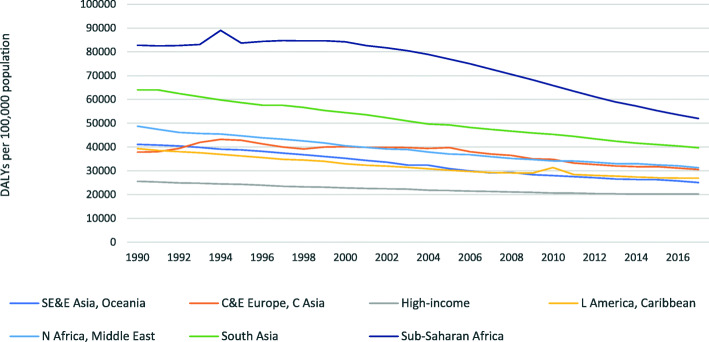
DALY rates by GBD world region, 1990–2017

The interpretation of *P* depends on whether aggregate DALY rates are growing or declining. Figure 4 illustrates that DALY rates have generally declined over the period 1990–2017. A positive (negative) *P* value indicates that declines in the *DALY rates from the high-ranked — ‘major’ (low-ranked — ‘minor’) — causes are disproportionately responsible for the declining aggregate rates*.^4^ For our example above, a positive *P* is combined with reduced DALY rates, meaning that the major diseases were disproportionally responsible for the declines of the disease burden.

Each of the potential interpretations of the sign of the proportionality index are summarized in Table 1. Because DALY rates have generally been in decline, these interpretations are indicated in bold. In addition to interpreting the sign associated with the proportionality indices, we refer the interested reader to Appendix 4 for an interpretation of their magnitudes. The practical use of the Gini coefficient and its decomposition lies in the comparison between regions and over time.^5^

**Table 1:** Interpretation of the Jenkins-Van Kerm (JVK) proportionality index

Aggregate DALY rate	Sign of proportionality (*P*) index	Causes disproportionately responsible for growth/decline
Growing	Positive	Low-ranked
	Negative	High-ranked
**Declining**	**Positive**	**High-ranked**
	**Negative**	**Low-ranked**

The reranking index, *R*, now gives an indication of the importance of the change in ranks of disease burden causes. It therefore summarizes the ‘mobility’ and stability of disease causes. *When diseases do not change ranks over time, the reranking index R equals 0 and it increases when more reranking takes place.* To illustrate the interpretation of *P*, *R*, and the Gini coefficient, consider the possible reasons for a small change in Gini (concentration of disease burden) over time. First, substantial proportionality (high level of *P*) can be offset by substantial reranking (a high *R*). That is, while the major diseases are disproportionally responsible for the decline in disease burden, the concentration of disease burden remains similar if there is substantial reranking over time. Second, a small change in concentration may be caused by both low proportionality and reranking.

### Data and presentation

The data used are taken from the 2017 GBD study which is publicly available and can be accessed by the query tool on the Institute for Health Metrics and Evaluation (IHME) website. Annual estimates of DALY burdens are available from 1990 to 2017, for 195 countries and 290 causes of DALYs [12]. 6

To better illustrate our main results in the tables, the analyses are complemented with information for individual diseases obtained from the GBD query tool and GBD Compare. This information is used in the main text to clarify the analyses, but they are not the focus of this paper. In particular, DALY rates are provided alongside the decomposition indices to facilitate the interpretation of the proportionality indices. The number of causes that are used in each decomposition calculation is presented in brackets in each table.

Colour shading indicates the relative size of the *P* indices. The range of values used to determine the percentile-based colour shading is determined by the P index values presented in each table, so it is not consistent with shading in other tables. All computations were done using age-standardized DALY rates, as is appropriate in order to better account for the differences in age structures across the world and the changing age structures within regions over time [1].^7^ Moreover, rates rather than crude totals were used to adjust for population changes in the regions over time.

## Results

Table 2 shows Gini coefficients, Gini changes and their decompositions presented across the 28 available years of data, for three 9-year periods, across the 7 GBD world regions, and for 290 DALY causes.

**Table 2:**
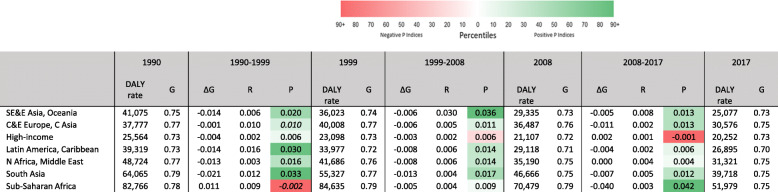
All DALY causes, by GBD world region. Gini coefficients and DALY rates, 1990, 1999, 2008, 2017; 9-year Gini changes and reranking and proportionality indices, 1990–1999, 1999–2008, 2008–2017

Along with DALY rates, Gini coefficients have generally fallen over the period. This is the result of disproportionate drops among the major causes. The table summarizes changes in the relative importance of CoD. We focus here on two regions where rather dramatic changes occurred. First, for the period from 1990 to 1999, *Sub-Saharan Africa* experienced increases in both overall DALY rates, and in the Gini coefficient. Underlying this are high reranking and negative proportionality indices which are primarily are result of the rapid development of the HIV/AIDS epidemic during this period. HIV/AIDS first overtook malaria, then diarrheal diseases, then lower respiratory infections, and by 1999 it had become the leading cause of DALYs. In sharp contrast to this, the large positive proportionality index for the 2008 to 2017 period signals the steep falls in the HIV/AIDS DALY rate, as well as for malaria, diarrheal diseases, and lower respiratory infections. Secondly, the period 1999 to 2008 shows relatively large-size and positive *R* and *P* indices in *South East Asia, East Asia, and Oceania*, combined with a particularly steep drop in total DALY rates. This is primarily caused by the sharp fall for two of the leading causes of DALYs from 1999: chronic obstructive pulmonary disease and lower respiratory infections. These declines also led to reductions in their rankings which, in turn, led to IHD, intracerebral haemorrhage, and stroke regaining their former places in the rankings.

While the retrospective information on longer-term trends is of interest, for the purpose of aiding policymakers in making investment and resource reallocation decisions, we now adopt a shorter-term view. In Table 3, Gini coefficients, reranking, and proportionality indices are presented for the 2017 and 2007. Alongside these 10-year decompositions, year-on-year proportionality and reranking indices are presented, allowing for a more detailed inspection of the changes occurring in this period.

**Table 3:**
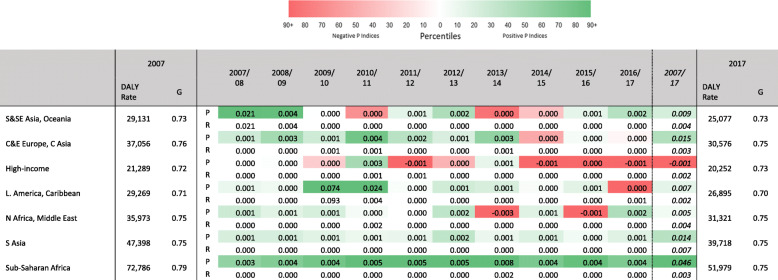
All DALY causes, by GBD world region. Gini coefficients and DALY rates, 2007 and 2017; yearly proportionality and reranking indices, 2007/2008–2016/2017; 10-year reranking and proportionality indices, 2007–2017

During this decade, the large decline in HIV/AIDS and, to a lesser extent, in malaria and tuberculosis, is responsible for the observed trends in the proportionality indices for Sub-Saharan Africa. While such trends are less clear for other regions, some outliers are discernible. The 2009/2010 Latin America and Caribbean and the 2007/2008 South East Asia, East Asia, and Oceania proportionality indices correspond to the 2010 Haiti and 2008 Sichuan earthquakes, respectively [23, 31]. The figures are large in magnitude, and italicized, which indicates that there were rises in total DALYs during those years, and that low-ranked causes, especially *‘Exposure to the forces of nature’* were disproportionately responsible for these. This cause also influences the reranking index since it is a major cause in 1 year and a minor cause in all other years.

More generally, regions experienced falls in rates of disease burden (see Fig. 2). Table 3 indicates that these trends correspond to a general reduction in proportionality indices and, in some cases, to negative *P* indices, especially since 2013. This means that the falls in rates of disease burden in most regions were increasingly due to disproportionate falls among lower ranked causes. Over time, it can be seen that in North Africa and the Middle East, much as in Sub-Saharan Africa, the size of the proportionality index is quite high in several years. This signals that there are substantial changes in the relative importance of diseases. This finding is likely explained by conflict and violence in North Africa and the Middle East.

In Tables 4, 5, and 6 the Gini coefficients and decompositions are presented for the groups of disease burden causes defined by the GBD.^8^ This method has the advantage of allowing proportionality indices to show whether or not DALY rates are becoming more concentrated in the major causes within a particular group of disease causes. Reranking indices represent the reranking of causes within groups of causes.

**Table 4:**
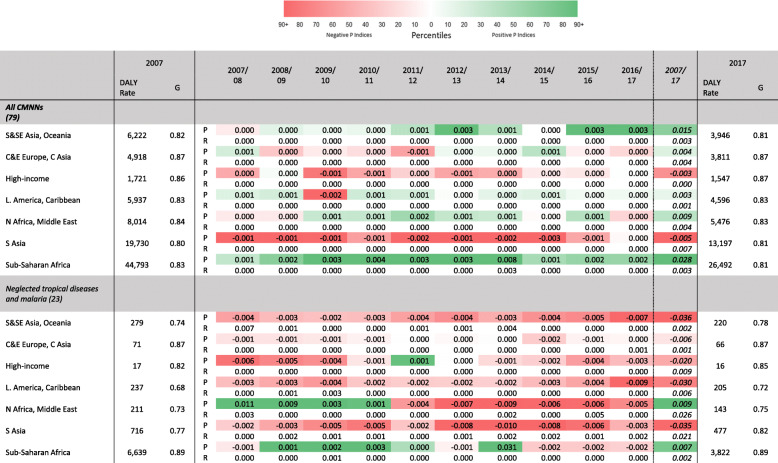
CMNN disease-specific DALY causes, by GBD world region. Gini coefficients and DALY rates, 2007 and 2017; yearly proportionality indices, 2007/2008–2016/2017; 10-year reranking and proportionality indices, 2007–2017

**Table 5:**
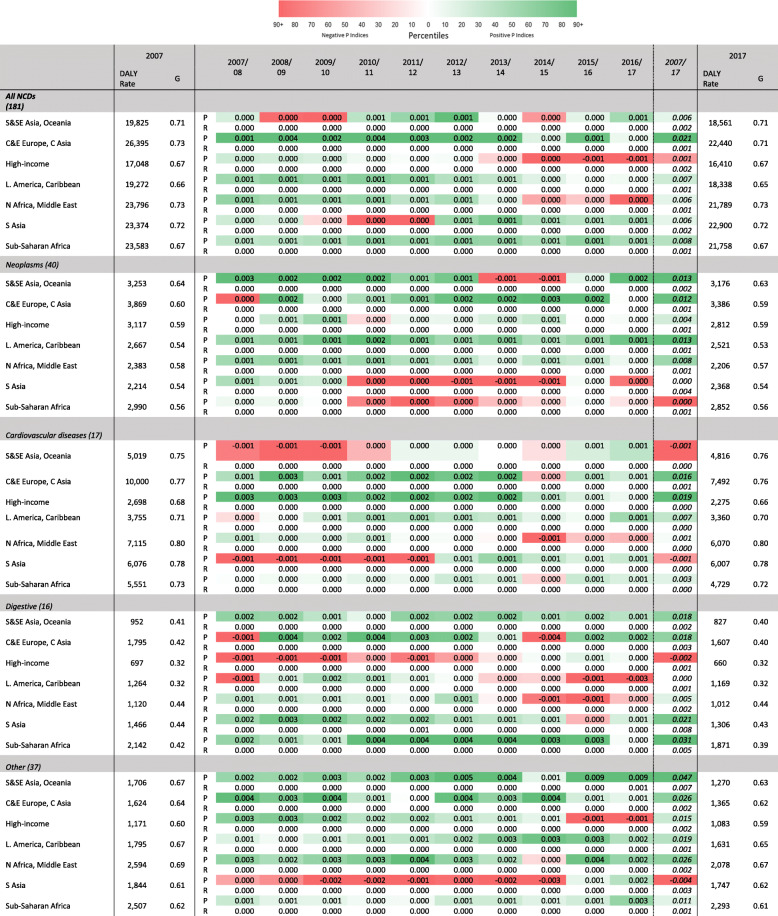
NCD-specific DALY causes, by GBD world region. Gini coefficients and DALY rates, 2007 and 2017; yearly proportionality indices, 2007/2008–2016/2017; 10-year reranking and proportionality indices, 2007–2017

**Table 6:**
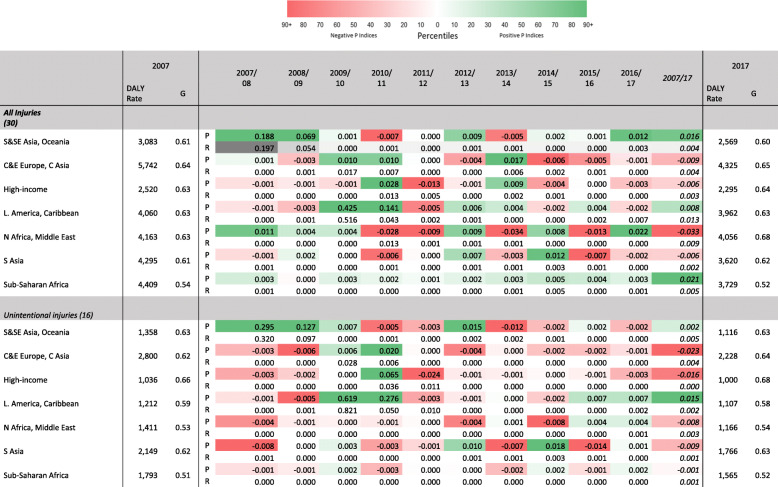
Injury-specific DALY causes, by GBD world region. Gini coefficients and DALY rates, 2007 and 2017; yearly proportionality and reranking indices, 2007/2008–2016/2017; 10-year reranking and proportionality indices, 2007–2017

Table 4 presents Gini coefficients, reranking, and proportionality indices for the groups of disease burden causes defined in the GBD as CMNN diseases. While sub-Saharan Africa and S&SE Asia have seen their burden of disease decline because of major CMNN diseases, the opposite is true for other world regions. Especially the high-income and South Asia region experienced relatively large declines in disease burden of minor diseases. The positive proportionality indices and falling DALY rates for the *HIV/AIDS and STIs* category in sub-Saharan Africa indicate that the cause *HIV/AIDS resulting in other diseases* fell far more steeply than other causes within that category. The South Asia region shows the second highest CMNN DALY rate after sub-Saharan Africa. The negative proportionality indices for this region within the *Neglected tropical diseases and malaria* category reflect the rises in DALY rates from dengue fever, which appear to outweigh the reductions in DALY rates from malaria.

Table 5 presents Gini coefficients and reranking and proportionality indices for the groups of causes defined in the GBD as NCDs. When considering NCDs as a whole, progressivity and reranking indices display very low values. Other NCD causes contribute less to the overall DALY rate but, nonetheless, there is a relatively high-magnitude and positive *P* index for *Neoplasms* in Central and Eastern Europe. This may signal the steeper drops in lung and stomach cancer DALY rates relative to other cancers. Table 6 presents results for the causes defined as injuries. In contrast to CMNNs and NCDs, no clear trend can be discerned among injuries. Most remarkable are the substantial *P* indices due to the 2010 Haiti and 2008 Sichuan earthquakes. These also explain the overall *P* indices but are more pronounced when restricting attention to injuries.

## Discussion and limitations

The relevance of our suggested measures is apparent from the results for the period 1999 to 2008. For example, during this period, *R* and *P* indices were particularly large in South East Asia, East Asia, and Oceania. However, in spite of these large and important changes, the change in the overall Gini coefficient is almost negligible and does not reveal the underlying changes. Therefore, this example illustrates the usefulness of the decomposition for identifying changes in the relative importance of causes.

Through year-on-year comparisons of proportionality indices, we found that minor diseases are becoming more important in explaining the declining disease burden. It is likely that the decreased rate of reductions in DALY rates due to IHD and the continuing rise in importance of causes such as Alzheimer’s disease, especially in high-income countries, are among the most important contributors to this trend. The relative importance of already high-ranked causes has been rising in recent years because DALY rates for these causes have fallen at a slower rate than for minor causes. This observation could justify more resources being reallocated to the corresponding types of health care interventions. However, the small size of the reranking indices suggests that resources should not be reallocated in a way that allows for the amount of resources allocated to lower ranked causes to overtake that of the higher ranked. At the regional level, the large proportionality indices for Sub-Saharan Africa signal that the relative importance of diseases is quite variable over time. The best way forward for investments and resource allocation seems to be to target multiple CoD burden in order to best mitigate the risks associated with future uncertainty.

Cause-specific analyses suggest that the relative importance between disease causes is rising most for CMNN diseases, which is demonstrated by their indices being generally higher than for NCDs. For most NCDs in most regions, the proportionality indices are either relatively constant, or falling in more recent years. This is likely to reflect the effect of a slowing down in the reduction of IHD disease burden. This is confirmed by the results for the *Cardiovascular diseases* category.

Our study has limitations. First, while the proportionality index is useful to identify which CoD burdens are changing in importance relative to one another, its value will be close to zero if there are no changes in relative importance. This means that readers should be careful to note that just because the value of the index is low; this does not mean that there are no changes in the aggregate DALY rates, i.e. DALY rates could be rising or falling at the same rates for all causes. It is therefore advisable, as is done in our tables, to view the index in conjunction with changes in aggregate rates. Second, we provide summary measures to interpret extensive amounts of data. Of course, the interpretation of these measures still needs scrutiny of the underlying data to evaluate what is driving the change in these measures to inform policy. Third, there is uncertainty in the GBD estimates, and the GBD provides the 95% confidence intervals. For the purposes of this paper, only the central estimate has been used.

## Conclusion

The findings presented here demonstrate the usefulness of the Gini decomposition as a way of summarizing the data on trends for the large number of disease burden causes. It has a major advantage which no current method of summarizing the data manages to overcome: no matter how many of the 290 CoD burden are included in its calculation, it can summarize in a single statistic whether or not the leading CoD burden are rising or falling in importance, and whether any significant reranking is taking place.

For every region of the world, more recent years have witnessed lower — and in some cases negative — values of proportionality indices combined with a general deceleration in the rate of falls in disease burden rates. This finding implies that the rate of decline in the rates of disease burden of the leading causes has slowed relative to that of lower ranked causes.

The condensed nature of the presented data allows readers to more easily discover whether, for particular world regions, countries, or groups of causes, the leading CoD burden are becoming more or less important relative to lower ranked causes. For policymakers, the use of this summary measure could help to decide whether resources need to be reoriented to meet such a challenge.

## Figures and Tables

**Table 8:**
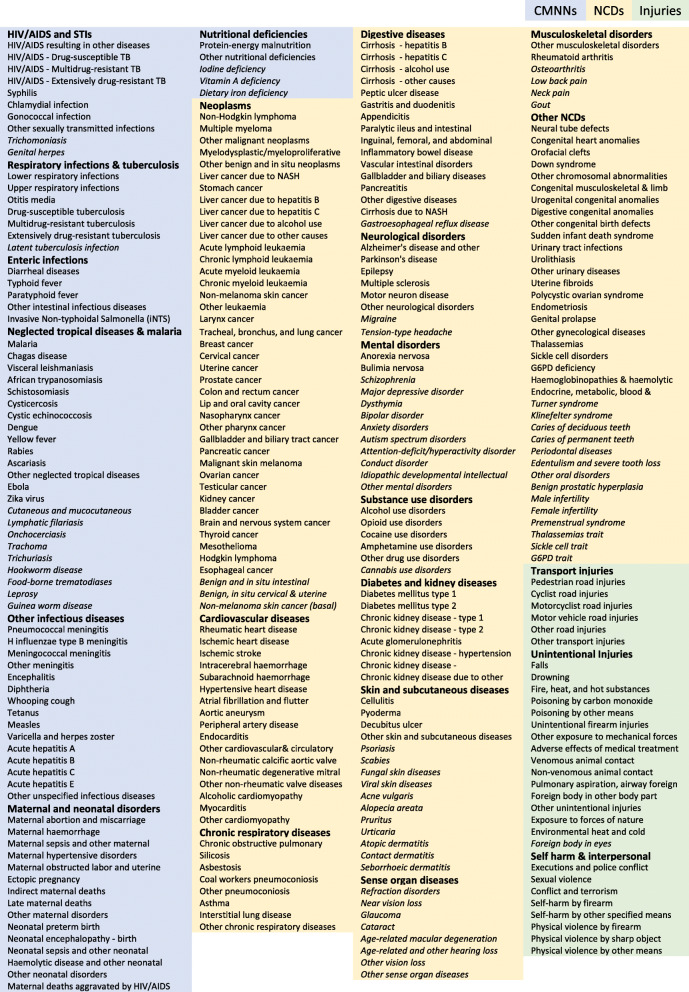
Causes of DALYs in the GBD

Italicized causes are classified as DALY causes but not death causes within the GBD

## Data Availability

Data are publicly available from http://ghdx.healthdata.org/gbd-results-tool.

## Appendix 1

The Gini is equal to one minus twice the area under the Lorenz curve and is formally defined as

4 $$ G=1-2{\int}_0^1L(s) ds $$

where *G* is the Gini coefficient and *L* is the Lorenz curve, which itself is a function of *s*, the cumulative distribution function of the disease causes [22].

## Appendix 2

This appendix provides a short proof for the result seen in Eq. (1), following from Jenkins and van Kerm [15].

Letting *G*_0_ and *G*_1_ be the Gini coefficients in years 0 and 1, then

$$ \Delta  G={G}_1-{G}_0 $$

Therefore, using Eq. (4)

$$ {\displaystyle \begin{array}{l}\Delta  G=1-2{\int}_0^1{L}_1(s) ds-\left[1-2{\int}_0^1{L}_0(s) ds\right]\\ {}=2{\int}_0^1{L}_0(s)-{L}_1(s) ds\end{array}} $$

Let ${C}_1^{(0)}(s)$ be the concentration curve of year 1 ordered according to year 0 ranks. Adding and subtracting from the above equation:

5 $$ {\displaystyle \begin{array}{l}\Delta  G=2{\int}_0^1{L}_0(s)-{L}_1(s)+{C}_1^{(0)}(s)-{C}_1^{(0)}(s) ds\\ {}=2{\int}_0^1{C}_1^{(0)}(s)-{L}_0(s) ds-2{\int}_0^1{C}_1^{(0)}(s)-{L}_1(s) ds\\ {}=R-P.\end{array}} $$

## Appendix 3

By integrating by parts and applying a change of variable, *s* = *F*(*x*), the equations in this paper for the Gini coefficients, their changes over time, and the decomposition of these changes, can be reformulated to demonstrate the roles of the mean DALY rates over all causes, the rankings of the individual causes, and the ‘proportional’ rates (i.e. the proportion of total DALY rates that each individual cause is responsible for). In this way, Eq. (3) can be reformulated to show that [15]

6 $$ P=2{\iint}_{z_{-}}^{z_{+}}w\left(F\left({x}_0\right)\right)\left[\frac{x_1}{T_1}-\frac{x_0}{T_0}\right]h\left({x}_0,{x}_1\right)d{x}_0d{x}_1 $$

In Eq. (6), *x*_*i*_ and *T*_*i*_ represent the DALY rates for each DALY cause in year *i*, and the total DALY rate in year *i*. *F*(.) is the cumulative density function of the DALY causes and *h*(.) denotes the joint probability density function of the DALY causes in years 0 and 1. *z*_+_ and *z*_−_ show the upper and lower limits of the domain of *x*_0_ and *x*_1_, so that *z*_+_ = *F*^−1^(1) and *z*_−_ = *F*^−1^(0).

There are two key points to note about this equation. Firstly, *F*(*x*_0_) is the proportion of DALY causes with a DALY rate less than *x*_0_ and can therefore be considered the ranking for each cause. Secondly, the weight *w*(.) is a decreasing function of *F*(.). More specifically, *w*(*F*(*x*)) = 2(1 − *F*(*x*)) so that lower ranked causes (causes responsible for fewer DALYs) are attributed a higher weight.

In summary, the formula shows that the proportionality index, *P*, should be thought of as the weighted average of the changes in proportional DALY rates between years 0 and 1 with the weights being determined by the rankings in year 0.

To relate the above equation to the interpretations made in Table 1, it is best to reformulate the above equation as follows:

Let $\pi =\frac{T_1-{T}_0}{T_0}$ be the proportional change in the total DALY rate. Also, let a generalized Kakwani [16]-type index be represented by:

7 $$ K=2{\iint}_{z_{-}}^{z_{+}}w\left(F\left({x}_0\right)\right)\left[\frac{x_1-{x}_0}{T_1-{T}_0}-\frac{x_0}{T_0}\right]h\left({x}_0,{x}_1\right)d{x}_0d{x}_1 $$

Then,

8 $$ P=\frac{\pi }{1+\pi }K. $$

*To obtain a positive proportionality index*: If *π* > 0, *P* is positive only if *K* is also positive. Due to the greater weights allocated to lower ranked causes, growth in DALY rates among these causes must be high relative to higher ranked causes for *K* to be positive. Conversely, if *π* < 0, then reductions in DALY rates among the lower ranked causes must be low relative to higher ranked causes for *K* to be negative.

*To obtain a negative proportionality index:* If *π* > 0, *P* is negative only if *K* is negative. Due to the greater weights allocated to lower ranked causes, growth in DALY rates among these causes must be low relative to higher ranked causes for *K* to be negative. Conversely, if *π* < 0, then reductions in DALY rates among the lower ranked causes must be high relative to higher ranked causes for *K* to be positive.

## Appendix 4

Blackburn [3] proposed the use of a simple formula to interpret Gini changes. The equivalent version of this formula outlined by Van Doorslaer and Koolman [25] is as follows:

9 $$ k=200\Delta  G $$

For declines in the Gini coefficient, *k* in Eq. (9) represents the percentage of the average DALY rate that would need to be equally redistributed as a lump sum from above-median to below-median DALY causes for the Gini in year 0 to be reduced to its year 1 level.^9^ For growth in the Gini coefficient, this redistribution would need to be from below-median to above-median causes.

However, Van Doorslaer and Koolman [25] clarify that the above interpretation only applies if rankings are held constant to their pre-distribution position. Therefore, it follows that if rankings do change over the period of interest then Eq. (9) does not hold. However, given our earlier definition of the progressivity index, the implication is that:

10 $$ k=200P $$

Therefore, the approach suggested by Blackburn [3] can be used to help interpret the size of progressivity indices.

## Appendix 5

**Table 7:** Countries and territories by GBD world region

SE&E Asia, Oceania	C&E Europe, C Asia	High-income	L America, Caribbean	N Africa, Middle East	South Asia	Sub-Saharan Africa
Southeast Asia, East Asia, and Oceania	Central Europe, Eastern Europe, and Central Asia	High-income	Latin America and Caribbean	North Africa and Middle East	South Asia	Sub-Saharan Africa
China	Armenia	Brunei	Antigua and Barbuda	Algeria	Bangladesh	Angola
North Korea	Azerbaijan	Japan	The Bahamas	Bahrain	Bhutan	C. African Republic
Taiwan	Georgia	South Korea	Barbados	Egypt	India	Congo
Cambodia	Kazakhstan	Singapore	Belize	Iran	Nepal	DR Congo
Indonesia	Kyrgyzstan	Australia	Cuba	Iraq	Pakistan	Equatorial Guinea
Laos	Mongolia	New Zealand	Dominica	Jordan		Gabon
Malaysia	Tajikistan	Andorra	Dominican Republic	Kuwait		Burundi
Maldives	Turkmenistan	Austria	Grenada	Lebanon		Comoros
Myanmar	Uzbekistan	Belgium	Guyana	Libya		Djibouti
Philippines	Albania	Cyprus	Haiti	Morocco		Eritrea
Sri Lanka	Bosnia and Herzegovina	Denmark	Jamaica	Palestine		Ethiopia
Thailand	Bulgaria	Finland	Saint Lucia	Oman		Kenya
Timor-Leste	Croatia	France	St Vincent, Grenadines	Qatar		Madagascar
Vietnam	Czech Republic	Germany	Suriname	Saudi Arabia		Malawi
Fiji	Hungary	Greece	Trinidad and Tobago	Syria		Mauritius
Kiribati	Macedonia	Iceland	Bolivia	Tunisia		Mozambique
Marshall Islands	Montenegro	Ireland	Ecuador	Turkey		Rwanda
Micronesia	Poland	Israel	Peru	United Arab Emirates		Seychelles
Papua New Guinea	Romania	Italy	Colombia	Yemen		Somalia
Samoa	Serbia	Luxembourg	Costa Rica	Afghanistan		Tanzania
Solomon Islands	Slovakia	Malta	El Salvador	Sudan		Uganda
Tonga	Slovenia	Netherlands	Guatemala			Zambia
Vanuatu	Belarus	Norway	Honduras			Botswana
American Samoa	Estonia	Portugal	Mexico			Lesotho
Guam	Latvia	Spain	Nicaragua			Namibia
N. Mariana Islands	Lithuania	Sweden	Panama			South Africa
	Moldova	Switzerland	Venezuela			Swaziland
	Russian Federation	UK	Brazil			Zimbabwe
	Ukraine	Argentina	Paraguay			Benin
		Chile	Bermuda			Burkina Faso
		Uruguay	Puerto Rico			Cameroon
		Canada	Virgin Islands, USA			Cape Verde
		USA				Chad
		Greenland				Cote d’Ivoire
						The Gambia
						Ghana
						Guinea
						Guinea-Bissau
						Liberia
						Mali
						Mauritania
						Niger
						Nigeria
						Sao Tome and Principe
						Senegal
						Sierra Leone
						Togo
						South Sudan

## Abbreviations

CMNN: Communicable, maternal, neo-natal and nutritional diseases
DALY: Disability Adjusted Life Year
*G*: Gini index
GBD: Global Burden of Disease
IHD: Ischemic heart disease
IHME: Institute for Health Metrics and Evaluation
JVK: Jenkins-Van Kerm index
NCD: Non-communicable diseases
*P*: Proportionality index
*R*: Reranking index
S&SE: South and South-East
STI: Sexually transmitted infection
WHO: World Health Organization

## Footnotes

1 DALYs are defined as the sum of years of life lost due to premature death (YLLs) and the years of life lived with a disability (YLDs). See GBD study [10].

2 See Appendix 3 for a demonstration of this result; *P* refers to progressivity in Jenkins and Van Kerm, but the term proportionality is more applicable here.

3 Gini and concentration indices can be estimated with the sgini or conindex commands in the software package Stata. More generally, they can be obtained from convenient covariance or regression approaches. These practical steps are intuitively described in [22], chapter 8.

4 A mathematical exposition for these interpretations can be found in Appendix 2.

5 Many papers on income inequality and socioeconomic inequality in health compare Gini coefficients and Concentration Indices between places and over time. See, e.g. Van Ourti et al. [26] for an example.

6 An overview of countries, world regions, and diseases is provided in Tables 7 and 8 in Appendix 5.

7 Statistics Canada provides an explanation of age-standardization of mortality rates: https://www.statcan.gc.ca/eng/dai/btd/asr.

8 Where a group contains fewer than 15 death causes, this group is excluded from the tables. The number of causes within a group are shown in brackets within each table.
